# Chronic myeloid leukemia: the paradigm of targeting oncogenic tyrosine kinase signaling and counteracting resistance for successful cancer therapy

**DOI:** 10.1186/s12943-018-0780-6

**Published:** 2018-02-19

**Authors:** Simona Soverini, Manuela Mancini, Luana Bavaro, Michele Cavo, Giovanni Martinelli

**Affiliations:** Hematology/Oncology “L. e A. Seràgnoli”, Department of Experimental, Diagnostic and Specialty Medicine, University of Bologna, Bologna, Italy

**Keywords:** BCR-ABL1, Tyrosine kinase, Tyrosine kinase inhibitors, Resistance

## Abstract

Deregulated activity of BCR-ABL1, a nonreceptor tyrosine kinase encoded by the fusion gene resulting from the t(9;22)(q34;q11) chromosomal translocation, is thought to be the driver event responsible for initiation and maintenance of chronic myeloid leukemia (CML). BCR-ABL1 was one of the first tyrosine kinases to be implicated in a human malignancy and the first to be successfully targeted. Imatinib mesylate, the first tyrosine kinase inhibitor (TKI) to be approved for therapeutic use, was hailed as a magic bullet against cancer and remains one of the safest and most effective anticancer agents ever developed. Second- and third-generation TKIs were later introduced to prevent or counteract the problem of drug resistance, that may arise in a small proportion of patients. They are more potent molecules, but have been associated to more serious side effects and complications. Patients achieving stable optimal responses to TKI therapy are predicted to have the same life expectancy of the general population. However, TKIs do not ‘cure’ CML. Only a small proportion of cases may attempt therapy discontinuation without experiencing subsequent relapse. The great majority of patients will have to assume TKIs indefinitely – which raises serious pharmacoeconomic concerns and is now shifting the focus from efficacy to compliance and quality of life issues. Here we retrace the steps that have led from the biological acquisitions regarding BCR-ABL1 structure and function to the development of inhibitory strategies and we discuss drug resistance mechanism and how they can be addressed.

**Electronic supplementary material:**

The online version of this article (10.1186/s12943-018-0780-6) contains supplementary material, which is available to authorized users.

## Introduction

Chronic myeloid leukemia (CML) is a rare disease worldwide: its incidence is estimated to be 1–2 cases/100,000/year [1]. However, the advances in biology and therapy of CML have set gigantic milestones in the history of anticancer precision medicine. CML has been the first human malignancy to be associated, almost 60 years ago (well before the ‘omics’ era!) to a consistent chromosomal abnormality. Between the 60s and the 90s, a series of seminal studies clarified that the deregulated activity of a tyrosine kinase, BCR-ABL1, resulting from that chromosomal abnormality, seemed to be necessary and sufficient to induce leukemia. As a consequence, CML became the first human malignancy for whom the ‘dream’ of targeted therapy could come true. The tyrosine kinase inhibitor (TKI) imatinib mesylate was approved for resistant/refractory CML patients in 2001, and for newly diagnosed patients just two years later. Cases of acquired resistance to imatinib, however, began to be reported soon after the first clinical trials commenced – temporarily casting shadows over the long-term efficacy of targeted therapies: might CML and cancer in general be a more tougher enemy than initially expected? Many years later, further biological and clinical advances have led to three generations of TKIs, to a life expectancy for CML patients approaching that of the general population and to the possibility to safely and permanently stop therapy in a small but significant proportion of cases - although the issue of drug resistance is not yet fully solved. This review summarizes the main biological acquisitions about BCR-ABL1 as a therapeutically druggable oncogenic tyrosine kinase and provides an update on drug resistance mechanisms and how they can be overcome.

### CML: The disease

**Fig. 1:**
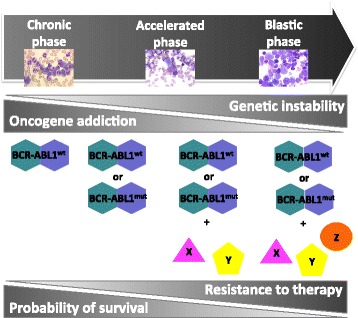
Progression of CML from chronic phase (CP) to blastic phase (BP). Biologically, the transition is associated with the accumulation of additional hits in *BCR-ABL1* itself (TKI-resistant kinase domain mutations) or in other genes/chromosomes. In the latter case, the degree of oncogenic addiction decreases, and inhibiting BCR-ABL1 alone may not be sufficient any more. This translates into an increase of drug resistance and in poor response to current therapies. ‘X’, ‘Y’ and ‘Z’ represent additional altered molecules other than BCR-ABL1

CML accounts for 15–20% of all cases of leukemia in adults [1]. Clinical hallmarks of CML are leukocytosis, a left shift in the differential count, and splenomegaly. The natural history of the disease follows a triphasic course with an initial chronic phase (CP), an intermediate accelerated phase (AP) and a final, fatal blastic phase (BP)(Fig. 1). CP may last several years and is characterized by the expansion of the myeloid cell compartment, although cells still retain the capacity to differentiate and function normally. Symptoms in this phase are generally mild and many patients are asymptomatic, being often diagnosed incidentally after a routine blood test. AP, that may have a variable duration from weeks to years and cannot always be recognized, is characterized by the appearance of more immature cells in the blood, frequent constitutional symptoms, and a less favorable response to therapy. The final stage is BP, where immature cells predominate and survival is measured in months. Progression from CP to BP is characterized by an increase in genetic instability leading to the accumulation of genetic/cytogenetic defects additional to the Ph chromosome and increased likelihood of drug resistance (Fig. 1). Although TKIs have greatly improved patient outcomes, up to 5% of patients may still progress from CP to BP and the prognosis of such patients remains quite poor [2]. Comprehensive catalogs of the additional genetic and functional defects observed in BP patients have been compiled [3, 4], but the mechanisms underlying disease progression have not been clarified yet.

Before the advent of targeted therapy, the gold standard for pharmacologic treatment was α-interferon (α-IFN), which was associated with a not negligible toxicity and a median survival time of approximately five years [5]; upfront allogeneic stem cell transplant was the only curative option. TKIs have revolutionized the life expectancy and quality of CML patients and have led to the introduction of the concept of ‘functional’ or ‘operational cure’ [6]. This is defined as avoidance of progression and resistance and durable freedom from any disease sign and symptom despite the possible presence of residual leukemic cells. At first, it was envisioned that functional cure could be achieved only with lifelong TKI treatment. More recently, however, several clinical trials have shown that 40 to 60% of the patients who achieve a deep and durable reduction or clearance of residual *BCR-ABL1* transcripts (‘Deep Molecular Response’) after several years of TKI treatment may safely interrupt their therapy without relapsing (‘Treatment-Free Remission’ (TFR); see [7–9] for detailed reviews on this issue, that is out of the scope of the present manuscript). Current clinical research is therefore focusing on avoiding resistance and increasing the rate of patients successfully achieving TFR.

### Structure and function of the BCR-ABL1 fusion tyrosine kinase

**Fig. 2:**
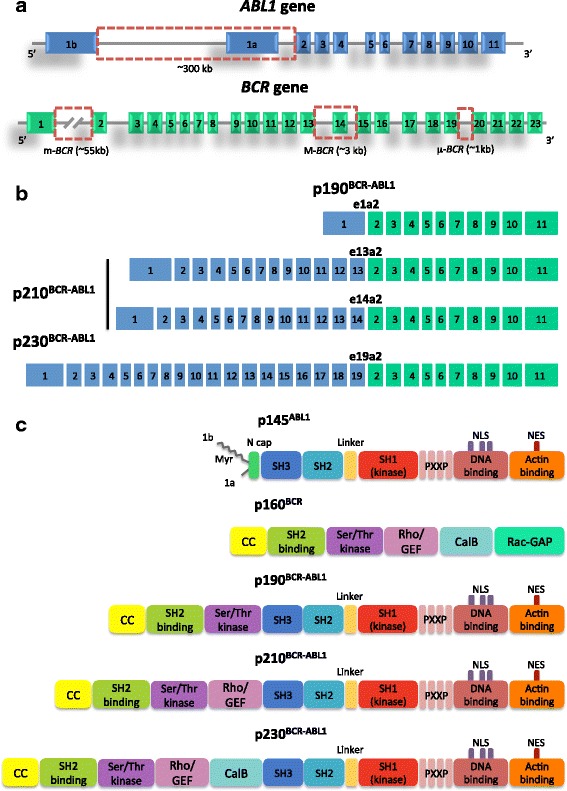
Genomic breakpoints in the *BCR* and *ABL1* genes and resulting transcript types and proteins. **a** Translocation breakpoints in *BCR* most frequently fall in intron 13 or 14 (M-*BCR*) or in intron 1 (m-*BCR*), or in intron 19 (μ-*BCR*). In *ABL1*, the breakpoints are intronic as well, and most frequently fall in a large region comprised between exons 1b and 2. Exon 1a and 1b are mutually exclusive and are incorporated in the mature *ABL1* mRNA as a result of alternative splicing. However, neither of the two is retained in *BCR-ABL1* mRNA. **b** The most common fusion transcripts resulting from the translocation include e13a2 and e14a2, resulting from the M-*BCR*, both translated into the p210^BCR-ABL1^ isoform (typical of CML and of some cases of Ph + ALL); e1a2, resulting from the m-*BCR* and translated into the p190^BCR-ABL1^ isoform (typical of the majority of Ph + ALL); e19a2, resulting from the μ-*BCR* and translated into the p230^BCR-ABL1^ isoform (typical of a subset of CML once called chronic neutrophilic leukemias). **c** Domain organization of BCR, ABL1 and BCR-ABL1 proteins. BCR is a 160 kDa protein with a coiled-coil (CC) oligomerization domain, a domain thought to mediate binding to Src-homology 2 (SH2)-domain-containing proteins, a serine/threonine kinase domain, a region with homology to Rho guanine-nucleotide-exchange factor (Rho-GEF), a region thought to facilitate calcium-dependent lipid binding (CaLB) and a region showing homology to Rac GTPase activating protein (Rac-GAP). ABL1 is a 145 kDa protein that contains an N-cap (that in isoform 1b undergoes myristoylation, a post translation modification that attaches the fourteen-carbon saturated fatty acid myristate to the amino-terminal glycine of the protein), the tandem SH3, SH2 and SH1 (tyrosine-kinase) domains, four proline-rich SH3 binding sites (PXXP), three nuclear localization signals (NLSs), one nuclear exporting signal (NES), a DNA-binding domain, and an actin-binding domain. In all BCR-ABL1 protein isoforms, the CC domain of BCR is included, the myristoylated N cap is lost, and the ABL1 kinase domain is retained. National Center for Biotechnology Information (NCBI) accession numbers: ABL1 gene, NG_012034.1; BCR gene, NG_009244.1

It was 1960 when a simple light microscope enabled Peter Nowell and David Hungerford to observe that a minute acrocentric chromosome was consistently detectable in the bone marrow cells of CML patients [10]. This chromosome was named ‘Philadelphia’ (Ph) after the city where its discovery took place. In 1973, once again just a microscope was enough for Janet Rowley to uncover that the Ph chromosome was the result of a reciprocal translocation between chromosomes 9 and 22: the t(9;22)(q34;q11) [11]. The subsequent leap forward came when the first molecular biology techniques became available. By the mid-1980s, it could be established that the t(9;22) translocation resulted in the juxtaposition, on the Ph chromosome, of Abelson 1 (*ABL1*), the human homologue of the v-abl oncogene carried by the Abelson murine leukemia virus (A-MuLV) located on the long arm of chromosome 9, to a gene of unknown function on the long arm of chromosome 22, which was called *BCR* for Breakpoint Cluster Region, since DNA breaks occurred in a relatively small genomic region [12, 13]. Association of the Ph chromosome with B-cell acute lymphoblastic leukemia (B-ALL) was also discovered [14]. A smaller 7.0 kb mRNA, as opposed to a CML Ph chromosome 8.5 kb mRNA product, was observed in B-ALL patients [15, 16]. Furthermore, the *BCR-ABL1* protein product in B-ALL samples was 185/190 kDa (p190^BCR-ABL1^) as opposed to the 210 kDa *BCR-ABL1* protein product (p210^BCR-ABL1^) detectable in CML samples [15, 17]. The differences in the Ph chromosome gene product in B-ALL versus CML were found to be the result of a different localization of *BCR* breakpoints: in B-ALL, they were mapped within the minor breakpoint cluster region (m-*BCR*) whereas in CML, they fell within the major breakpoint cluster region (M-*BCR*) (Fig. 2a). A third region where breakpoints may more rarely cluster is the so called μ-*BCR* (Fig. 2a). Depending on the breakpoint, and after alternative splicing, different *BCR-ABL1* transcripts may result (Fig. 2b). Further studies showed a high but not absolute correlation between the p210^BCR-ABL1^ form and CML, and between p190^BCR-ABL1^ and B-ALL, questioning whether specific forms of BCR-ABL1 may play a role in the aetiology of each leukemia. A p230^BCR-ABL1^ isoform (typical of a subset of CML once called chronic neutrophilic leukemia) resulting from the μ-*BCR* was later uncovered [18] (Fig. 2a-b). Over the years, additional, more rare fusion schemes have also been reported (Additional file 1: Figure S1).

Seminal was the discovery that the protein derived from the chimeric *BCR-ABL1* gene had tyrosine kinase activity, that derived from normal *ABL1* but was deregulated as a consequence of the translocation, and correlated with the ability to induce malignant transformation [19].

The BCR-ABL1 protein acquires some domains from BCR and others from ABL1 [20]. Domains from BCR include, depending on the genomic breakpoint position (Fig. 2c):

- an N-terminal coiled-coil (oligomerization) domain;
- a Serine/Threonine kinase domain containing a docking site (phosphorylated Tyrosine 177, Y177) for the adaptor protein growth factor receptor-bound protein 2 (GRB2);
- p210^BCR-ABL1^ also retains a Ras homolog gene family/Guanine nucleotide exchange factors (Rho/GEF) kinase domain;
- p230^BCR-ABL1^ additionally incorporates a Calcium-binding domain.

Domains from ABL1 include (Fig. 2c):

- three SRC homology domains (SH3, SH2, SH1) – the SH1 is the kinase domain, whereas the SH2 and SH3 domains mediate interactions with other proteins;
- a long C-terminal region of approximately 600 amino acids encoded by the last exon, which contains proline-rich sequences mediating the interaction of ABL1 with other SH3-containing proteins (like Crkl, an adapter molecule whose phosphorylation serves as readout for ABL1 kinase activation), a DNA-binding domain and an actin-binding domain. This region also contains nuclear localization and nuclear export signals regulating the nuclear-cytoplasmic shuttling of the kinase.

The reason why native ABL1 has a tightly regulated kinase activity whereas BCR-ABL1 shows constitutive activation lies essentially in the fact that BCR-ABL1 loses the the N-terminal “cap” (N-cap), a region with a signal sequence for myristoylation playing a critical regulatory role. The N-terminal myristic acid group binds a deep hydrophobic pocket in the C-terminal lobe of the kinase domain. Interaction of the myristoylated N-cap with the C-terminal lobe is critical to maintain an autoinhibited state. Loss of this region, together with fusion of BCR sequences encompassing the oligomerization domain and Y177, abrogate the physiologic control of the kinase.

The understanding of native ABL1 functions (recently reviewed in [21]) was the key to unravel how BCR-ABL1 may promote cellular transformation. The ABL1 protein is implicated in a wide range of cellular processes, including regulation of cell growth and survival, oxidative stress and DNA-damage responses, actin dynamics and cell migration, transmission of information about the cellular environment through integrin signaling. To this purpose, ABL1 interacts with several cellular proteins – including signalling adaptors, other kinases, phosphatases, cell-cycle regulators, transcription factors and cytoskeletal proteins. Overall, it seems that the ABL1 protein serves as a key hub that integrates signals from various extracellular and intracellular sources to control cell cycle and apoptosis. Two major mechanisms have been implicated in the malignant transformation by BCR-ABL1: a) altered adhesion to bone marrow stroma cells and extracellular matrix, and b) constitutively active mitogenic signaling and reduced apoptosis [22]. Several cellular cascades are hijacked by BCR-ABL1 to promote CML. They include the RAS/RAF/MEK/ERK pathway, the JAK2/STAT pathway, the PI3K/AKT/mTOR pathway (reviewed in [23]).

How slightly different BCR-ABL1 isoforms (p190^BCR-ABL1^ vs p210^BCR-ABL1^) can trigger such diverse diseases (CML has an indolent course and TKI therapy results in stable remissions in the great majority of cases; Ph + ALL is much more aggressive, responses to TKIs are not durable and prognosis is relatively poor) has long been under investigation. Besides the clearly different cell of origin, several studies over the years have addressed the issue of which pathways may be differentially activated by the two isoforms, up to two very recent quantitative comparative proteomic studies comparing their respective ‘interactomes’ and ‘phosphoproteomes’. [24, 25] Both studies showed, surprisingly, no differences in the extent of autophosphorylation and kinase activation. However, they identified differential interactions, differential signaling networks and also differential intracytoplasmatic localization [24, 25].

### The role of BCR-ABL1 in leukemogenesis: When one genetic hit is enough (?)

CML is considered a paradigm for precision medicine in that it is caused by a single deregulated protein that exhibits a ‘druggable’ gain of function and is expressed in leukemic cells but not in normal cells. The success of targeted therapy in CML has not yet been replicated in other malignancies since cancer is most frequently the result of stepwise accumulation of multiple genetic defects [26]. How can BCR-ABL1 be necessary and sufficient for disease initiation and maintenance? And is it really sufficient?

In vitro culture systems demonstrated that BCR-ABL1 can transform immature hematopoietic cells, some fibroblast cell lines, and hematopoietic cell lines rendering them growth factor-independent. In addition, several groups reported that a CML-like disease could be induced in mice transplanted with bone marrow infected with a *BCR-ABL1* retrovirus. In contrast, mutant isoforms of BCR-ABL1 carrying inactivating mutations in the SH1 domain, or mutants lacking the BCR coiled coil domain, did not induce leukemia. All these studies [27–30], conducted around the 90s, converged to demontrate that BCR-ABL1 is indeed the causative agent of CML and fostered the search for small molecule inhibitors. On the other hand, evidences have also been brought that challenge this view. There are marked strain differences in disease induction after *BCR-ABL1* retroviral expression, suggesting that the genetic background may influence the ability of the oncogene to initiate CML [29]. Even more interestingly, a conditional knock-in mouse in whom the human *BCR-ABL1* cDNA was knocked into the endogenous mouse *Bcr* locus so that it could be conditionally expressed with different tissue-specific Cre transgenes under the added control of the native *Bcr* regulatory elements, was found not to develop leukemia during its lifetime, despite expression of a constitutively active BCR-ABL1 tyrosine kinase was observed in the hematopoietic progenitors [31]. The authors thus postulated that i) physiologic BCR-ABL1 expression may be insufficient for development of a CML-like disease; ii) in the retroviral or transgenic models, non-physiologic, very high levels of BCR-ABL1 expression due to multiple copies of the oncogene and expression from a very active retroviral promoter, non-specificity of expression timing and locale and maybe also random insertion-site mutations could artificially select for disease development [31]. This study was published in 2013, but the idea that additional cooperating events might be required for the induction of CML was, indeed, not new. Between the 80s and the 90s, initial evidences were brought in support of the existence of a putative event preceding the acquisition of *BCR-ABL1* at least in a proportion of patients. Studies of X chromosome inactivation and glucose-6-phosphate dehydrogenase genotype had raised the hypothesis that clonal hematopoiesis might precede the acquisition of the Ph chromosome [32, 33]. In addition, starting from the 90s, five reports had been published about the detection of *BCR-ABL1* transcripts in circulating leukocytes of up to 65% of healthy individuals when using sensitive polymerase chain reaction (PCR)-based assays [34–38]. Overall, 380 samples have been analyzed in these studies. *BCR-ABL1* was detected in cord blood and newborns (up to 40%), children and adolescents (up to 56%), adults (20–59 yrs.; up to 65%) and elderly (> 60 yrs.; up to 65%). For unknown reasons, the e1a2 rearrangement (leading to p190^BCR-ABL1^) was much more frequently detected than the e13a2 or e14a2 rearrangements (leading to p210^BCR-ABL1^). It might be argued that in all the studies a nested reverse transcription (RT)-PCR strategy was used to enhance sensitivity, although such an approach has the known drawback of being more prone to contamination. Unfortunately, there is no follow-up information available for BCR-ABL1-positive cases. The latency period between acquisition of the Ph chromosome and overt clinical development of CML is unknown and it is likely to be highly variable. Atomic bomb survivors could develop CML up to 40 years later. On the other hand, there are reports of children > 1 year of age who were diagnosed with CML [39]. In spite of the technical issues, these data, together with case reports of patients with detectable Ph chromosome in their bone marrow cells but otherwise asymptomatic (with a follow-up of few years only, however) [40, 41] raise, among others, the hypothesis that other events are needed before a true malignant expansion can occur and overt CML may develop. Mathematical models predict that 2 or more genetic hits in the hematopoietic stem cells may be needed for CML to develop [42, 43]. Although CP CML has long been considered a genetically homogeneous entity, the power of next generation sequencing (NGS) is now changing this view. A few years ago, targeted NGS-based resequencing of the 25 most commonly mutated genes in myeloid leukemias/myelodysplasias revealed *ASXL1*, *TET2*, *RUNX1*, *DNMT3A*, *EZH2* and *TP53* mutations in 5 out 15 chronic phase CML patients at diagnosis [44]. In the same study, analysis of individual hematopoietic colonies showed that the great majority of mutations were part of the Ph + clone. However, targeted resequencing of subsequent samples during TKI treatment revealed that the *DNMT3A* mutation found in the Ph + cells of a patient at diagnosis was also present in the Ph- clone, implying that it preceded *BCR-ABL1* acquisition. [44] Now we know that *DNMT3A*, *TET2* and *ASXL1* mutations, among others, may indeed be found in healthy elderly individuals, where they correlate with the risk of hematologic cancer and all-cause mortality (‘CHIP’, clonal hematopoiesis of indeterminate potential) [45–47]. Such mutations are thought to represent the first hit, leading to a clonally expanded pool of pre-leukemic hematopoietic stem cells from which overt leukemia may subsequently evolve through the acquisition of additional, disease-shaping genetic lesions [48]. Most recently, a NGS-based screen of 92 myeloid-associated genes in 300 serial samples from 100 CP CML patients at diagnosis and after TKI therapy showed evidence of DNMT3A, TET2, ASXL1, BCOR and CREBBP mutations in both diagnosis and follow-up samples, despite response to TKI therapy and *BCR-ABL1 *transcript clearance [49]. This further indicates that up to 10% of CML patients may have CHIP-related mutations and reinvigorates earlier hypotheses of a multistep pathogenesis of CML – arising, at least in some cases, from pluripotent stem cells of a pre-existing Ph- clone that enjoys a growth advantage.

Prospective serial screening of healthy individuals to determine whether the presence of the *BCR-ABL1* oncogene in their blood predicts for future CML development would be of great interest. To this purpose, the use of digital PCR would enable to conjugate high sensitivity with a more precise and accurate count of *BCR-ABL1* transcripts. However, because CML occurs at a frequency of 1–2 cases per 100,000 per year, a very large cohort would be needed, together with analysis of an equal number of individuals without detectable *BCR-ABL1* transcripts.

### BCR-ABL1 inhibition strategies

**Fig. 3:**
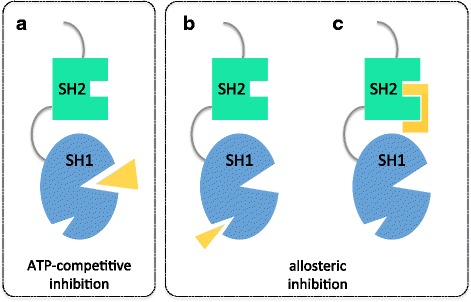
Stategies for BCR-ABL1 inhibition. Displayed are the SH2 domain (green) and the SH1 (kinase) domain (blue). The inhibitor is in yellow. **a** ATP-competitive inhibitors like imatinib, nilotinib, dasatinib etc. bind in the cleft between the N-lobe and the C-lobe, at the bottom of which lies the ATP-binding site. **b** One mode of allosteric inhibition is to use small molecules mimicking myristate binding to the hydrophobic pocket located in the C-lobe. This is the mode of action of asciminib. **c** Another mode of allosteric inhibition is to use proteins (‘monobodies’) directed against the SH2-kinase interface

Whether or not the only genetic (or epigenetic) hit, BCR-ABL1 is the main disease driver in CP CML, as testified by the remarkable clinical efficacy of TKIs. Based on the structural and functional features of BCR-ABL1, two inhibitory strategies have been devised. ATP-competitive inhibitors bind the kinase domain in the cleft between the N-terminal lobe and the C-terminal lobe. In contrast, allosteric inhibitors do not compete with ATP binding and rather bind to sites that are important regulators of kinase activity (Fig. 3).

#### ATP-competitive inhibitors

**Fig. 4:**
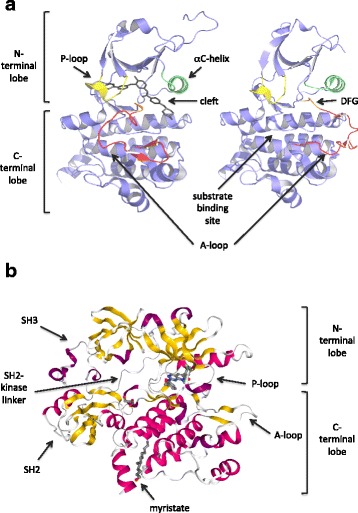
Regulation of the ABL1 tyrosine kinase. **a** All protein kinase domains have a highly conserved bilobed structure. The binding site for ATP and for the inhibitors is in a cleft between the 2 lobes. The phosphate-binding loop (P-loop) is highlighted in yellow. The phosphorylation state and conformation of the activation loop (A-loop; highlighted in red) determine whether the kinase is active or inactive. In all tyrosine kinases, the site of activating phosphorylation is generally a single Tyrosine residue located in the middle of the loop that once phosphorylated, can interact electrostatically with a neighboring Arginine residue, resulting in the stabilization of an extended and open conformation of the loop (right image). This conformation of the A-loop enables the access to the peptide substrate binding site. When the A-loop is unphosphorylated, it is folded inwards, blocking the peptide substrate binding site (left image). A second important regulatory feature of kinases is the conformation of a highly conserved aspartate-phenylalanine-glycine (DFG) motif (highlighted in orange) located at the N-terminal end of the A-loop. Images obtained with the Web-based 3D viewer NGL [113]. **b** Cartoon representation of ABL1 with the kinase domain (SH1), the SH2 and the SH3 domains. Alpha helices are in magenta, beta sheets in yellow. A myristic acid moiety in the myristate binding pocket is shown with a ball-and-stick representation. Binding of the myristoyl group to the myristate pocket induces a conformational change in the C-terminal helix of the kinase domain that is necessary for binding of the SH3-SH2 clamp, which keeps the kinase inactive. Image obtained with the web-based 3D viewer NGL [113] (Protein Data Bank [PDB] entry 1OPJ)

This is the first strategy that was historically pursued, with imatinib mesylate and its successors. Imatinib, originally designated ‘signal transduction inhibitor 571’ (STI571), arose from a time-consuming process of random screening of a library of thousands of compounds created using the structure of the ATP-binding site of protein kinase A. Imatinib is a 2-phenyl-amino-pyrimidine and it emerged as one of the most potent molecules inhibiting the ABL1 protein (although it also inhibits other kinases with even greater potency – the PDGFR family and c-KIT) [50]. The catalytic domains of all eukaryotic kinases have a highly conserved ‘dual lobe’ structure (Fig. 4a-b). The N-terminal lobe (residues 225–350 in ABL1) is made of five β-sheets and a single conserved α-helix, whereas the C-terminal lobe (residues 354–498 in ABL1) is helical. In the interface between the two lobes there is a cleft, where a series of highly conserved residues form the ATP-binding and catalytic sites. The activation state of kinases depends on the position of the so called ‘Activation loop’ (A-loop), a portion of the C-terminal lobe, which in ABL1 comprises amino acid residues 381–402 (Fig. 4a). In the active form of the kinase, the A-loop swings away from the catalytic center of the kinase (‘open’ conformation). The three N-terminal residues of the A-loop (amino acids 381–383) are a highly conserved D-F-G (Aspartate-Phenylalanine-Glycine) motif that is essential for catalytic activity (Fig. 4a). The C-terminal portion of the A-loop creates a platform for substrate binding. Although the conformation of the A-loop is highly conserved in kinases when they are in their active, open conformation, there are considerable differences in the inactive (closed) conformations. Kinases are usually activated by phosphorylation of key Serine/Threonine or Tyrosine residues within the A-loop. In the case of ABL1, Tyrosine 393 is phosphorylated and points away from the center of the kinase, allowing substrates to bind. In the inactive state of ABL1, Tyrosine 393 is unphosphorylated and points towards the center of the kinase, mimicking a substrate by forming a hydrogen bond with Asparagine 363. This occludes the mouth of the kinase, preventing substrates from binding. Crystal structure analysis of imatinib in complex with BCR-ABL1 showed that imatinib selectively binds to the inactive conformation of the kinase (type 2 inhibitor) (Additional file 2: Figure S2A). [51–53] Imatinib can trap the deregulated BCR-ABL1 oncoprotein once it transits through its inactive conformation. The resulting inhibition of BCR-ABL1 autophosphorylation and substrate phosphorylation blocks proliferation and induce apoptosis of CML cells. [54–56] Imatinib favourable oral bioavailability profile and the lack of significant toxicity in animal models led, starting from spring 1998, to a series of phase I and II clinical trials in patients with CP CML who had failed prior IFN-α and in patients with BP CML. The maximum tolerated dose was never achieved, adverse side effects were minimal (nausea, myalgia, edema, skin rash) and the rate of hematologic (normalization of blood cell count and differential, nonpalpable spleen) [57, 58] and cytogenetic (disappearance of the Ph chromosome in bone marrow metaphases) [57, 58] responses was truly remarkable. Taken together, these results established imatinib as a safe and effective therapy for all stages of CML and were the basis for the initial marketing approval by the Food and Drug Administration (FDA) on May, 2001, i.e., after less than 3 years from the start of the first phase I study. [59] On the same month, imatinib appeared on the cover of Time, hailed as ‘the magic bullet’ against cancer. After the first interim analysis of the phase III trial (the IRIS study – International Randomized Trial of Interferon and STI571; started in June 2000), in which the overwhelming superiority of imatinib over IFNα was rapidly consacrated (65% of the patients assigned to the IFNα arm crossed over to the imatinib arm mainly because of intolerance) [60], in December 2002, imatinib received the approval for first-line use in all newly diagnosed CML patients [61].

The problem of drug resistance (discussed below) and the fact many patients still had detectable *BCR-ABL1* transcripts in their blood and bone marrow at the minimal residual disease assessment, fostered the development of second- (and third-) generation TKIs (Table 1). Among the dozens and dozens of molecules that have been synthesized, tested in pre-clinical models and sometimes even in phase I trials, four only have successfully made all the way up to FDA and European Medicines agency (EMA) approval. Dasatinib is a thiazolylamino-pyrimidine emerged from a programme directed towards immunosuppressant drugs and, in addition to inhibiting the Src family kinases FYN, LCK, SRC and YES, it potently inhibits ABL1, c-KIT, PDGFRβ, EPHA2, HER1 and p38 MAP kinases [62]. Dasatinib is ~ 300-fold more potent than imatinib against BCR-ABL1 in vitro [63] and, unlike imatinib, is able to bind the open conformation (type 1 inhibitor)(Additional file 2: Fig. S2C) [64]. Nilotinib is a phenylamino-pyrimidine derivative structurally related to imatinib [65]. It was rationally designed based upon the crystal structure of imatinib-ABL1 complexes to enhance binding affinity and specificity, with less hydrogen bonds and more lipophilic interactions. As a result, nilotinib is 20- to 30-fold more potent than imatinib and is highly selective for BCR-ABL1. Nilotinib binds the inactive conformation of the kinase (type 2 inhibitor)(Additional file 2: Figure S2B), as imatinib does, but with a less stringent requirement in the absolute shape and charge of the binding surface of the protein. Bosutinib is an anilino-quinolinecarbonitrile that, like dasatinib, belongs to the class of dual SRC/ABL1 inihibitors and is a type 1 inhibitor (Additional file: Fig. S2D) [66]. In vitro, Bosutinib inhibits BCR-ABL1 with approximately 1-log greater potency as compared to imatinib [67]. All these second-generation TKIs have been shown in randomized clinical trials to induce faster and deeper molecular responses (logarithmic reduction in *BCR-ABL1* transcript levels) and reduce the number of cases who progress from CP to BP, as compared to imatinib. However, it is important to bear in mind that no significant differences in overall survival have yet emerged. Additionally, more severe adverse events and some serious complications have been reported with nilotinib (glucose elevation, liver and pancreatic enzyme elevation, CT prolongation, cardiovascular complications) and dasatinib (severe thrombocytopenias, pleural effusions, pulmonary arterial hypertension).

**Table 1:** List of approved ATP-competitive inhibitors and respective indications

First generation	
Imatinib	First-line and second/subsequent-line^a^, all disease phases
Second generation	
Dasatinib	First-line and second/subsequent-line, all disease phases
Nilotinib	First-line and second/subsequent-line, CP and AP only
Bosutinib	Third-line or when imatinib, dasatinib or nilotinib are not considered appropriate, all disease phases
Third generation	
Ponatinib	Second/subsequent-line if T315I+ or when no other TKI is considered appropriate, all disease phases

^a^being the weaker of all, imatinib is rarely considered as second/subsequent line option especially in case of resistance

Ponatinib is a third-generation TKI more recently developed to overcome the problem of the highly resistant T315I mutation, against whom all second generation TKIs remain ineffective (see below). It is a type 2 ABL1 inhibitor (Additional file 2: Figure S2E), also active against the SRC kinases and a number of receptor tyrosine kinases (KIT, RET, PDGFR, VEGF receptor, DDR, EPH, TRK, and FGFR family members) – indicating medium-range specificity (i.e., less specific than imatinib/nilotinib but more specific than dasatinib/bosutinib). Ponatinib resulted from a structure-guided drug design aimed to create a compound capable to bind the kinase domain irrespective of mutations (see below) [68]. Adverse events occurring during ponatinib treatment include thrombocytopenia, hypertension, lipase elevation and some severe complications like pancreatitis, arterial and venous thrombosis, heart failure have been reported at a rate that induced the FDA to end prematurely the phase III randomized study aiming at first-line registration.

#### Allosteric inhibitors

More recently, several allosteric regions in the BCR-ABL1 molecule have been identified and shown to be potentially druggable.

As anticipated above, the myristoylated N-cap of ABL1 plays a key role in kinase autoinhibition by binding a deep hydrophobic pocket in the C-terminal lobe. Binding of the myristoyl group to this pocket induces a conformational change in the C-terminal helix of the kinase domain that is necessary for binding of the SH3-SH2 clamp, which keeps the kinase inactive (Fig. 4b). This region is lost in BCR-ABL1, yet this control mechanism can be exploited by developing compounds that mimick myristate binding (Fig. 3b). GNF-2 [69] and GNF-5 are two such compounds. Clinical development of the first dropped mainly because of inefficacy against the T315I mutant. In contrast, the second (later renamed ABL001 or asciminib) is in advanced clinical development –phase II clinical trials are ongoing and a phase III randomized study of ABL001 versus bosutinib in chronic phase CML patients who have failed ≥2 TKIs has recently started. ABL001 and second-generation TKIs have similar cellular potencies but non-overlapping patterns of resistance mutations (see below), and combinations of both (Additional file 2: Figure S2F) might be the best strategy to prevent resistance in the first-line setting. Preclinical data are available about the combination of ABL001 and nilotinib [70].

Recent structural and functional studies have also highlighted the SH2-kinase interface as a key regulatory region with a stimulatory effect on kinase activity [71]. This interaction is thus another interesting target for pharmacologic interference. Although protein-protein interfaces were considered to be undruggable for a long time, the clinical use of the BH3-mimetic ABT-737 targeting Bcl-2 family members has led investigators to reconsider this old dogma in drug discovery. In recent studies, ‘monobodies’ were synthesized and tested [71, 72]. Monobodies are single-domain proteins, based on the fibronectin type III scaffold, that can be engineered to bind to a bait protein of choice with very high affinity. Monobodies engineered to bind a small cleft on the SH2 domain (Fig. 3c) inhibited BCR-ABL1 kinase activity in vitro and ex vivo, and they potently induced cell death in CML cell lines. In cell lines, delivery of the monobodies was achieved through lentiviral transduction/transfection. In vivo delivery of monobodies to target cells remains a challenge and safe and efficient routes of intracellular targeting will have to be devised for future therapeutic use of these molecules.

### Clinical resistance to BCR-ABL1 inhibitors: Mechanisms and frequency

It was 2001 and imatinib was still undergoing phase I-II trials when C. Sawyers’ group reported that BCR-ABL1 could escape from inhibition [73]. The analysis of a handful of patients with BP CML who had relapsed after an initial response had shown reactivation of BCR-ABL1 kinase activity despite continued imatinib treatment. A mechanism interfering with imatinib binding was hypothesized, and the entire kinase domain was sequenced in search of point mutations at some BCR-ABL1-imatinib contact residue. Strikingly, an identical substitution of Threonine to Isoleucine at residue 315 (T315I) was identified in six out of nine patients [73]. Initially, this finding casted a shadow over the long-term stability of responses to targeted therapy, since at that time it was difficult to predict how frequently such mutations would arise, thus neutralizing imatinib efficacy. Later on, however, it was realized that the earlier in the disease course TKI therapy is commenced, the lower is the relapse rate and the degree of genetic instability responsible for mutation acquisition. So, if TKI-resistant mutations remain, even nowadays, a challenge in patients with AP and BP, they arise much less frequently in CP patients who receive front-line TKI therapy [74]. In this setting, less than 30% of patients who fail therapy are found to harbor mutations (Soverini et al., unpublished).

Threonine 315 was later named ‘the gatekeeper’ residue, because it is strategically positioned to control the accessibility of the ATP-binding pocket. On binding, the hydroxyl group of Threonine 315 forms a hydrogen bond with imatinib, and the side chain present at position 315 also sterically controls the binding of the inhibitor to hydrophobic regions adjacent to the ATP-binding site [51, 75]. The substitution of Threonine with the bulkier and more hydrophobic Isoleucine was shown to eliminate this hydrogen bond, required for high-affinity inhibitor binding, and to create a steric hindrance interfering with imatinib placement [73, 75]. Notably, Threonine 315 is essential for imatinib binding but not for ATP binding. This means that the catalytic activity, hence the tumour-promoting function, is preserved in the imatinib-resistant T315I mutant. A strikingly identical amino acid substitution was later observed at homologous positions in the kinase domain of c-KIT (T670I) and PDGFRα (T674I) in imatinib-resistant gastrointestinal stromal tumors and hypereosinophilic syndromes, respectively [76, 77], further highlighting the central role of this highly-conserved ‘gatekeeper’ threonine in controlling the accessibility of the ATP-binding pocket. Accordingly, the T315I confers resistance to all the currently approved second-generation TKIs (dasatinib, nilotinib and bosutinib) and only the third-generation TKI ponatinib has demonstrated in vitro and in vivo activity against this mutant.

As the number of imatinib-resistant patients increased, sequencing of the kinase domain revealed a plethora of additional mutations. At present, more than 50 different mutation hotspots are known (Table 2). However, marked differences in IC_50_ values (the intracellular concentration of the drug required to inhibit by 50% proliferation or viability of a BaF3 cell line engineered to express a given BCR-ABL1 mutant) have been observed across these mutants, suggesting that the degree of insensitivity to imatinib may be variable [78]. Imatinib-resistant mutations have been detected at contact residues (F317L, Y253H), in the phosphate-binding loop (P-loop)(G250E, E255K), in the A-loop (H396R), and in other regions of the kinase domain where amino acid substitutions may possibly force the equilibrium towards the active conformation of the kinase, whom imatinib is unable to bind. In vitro sensitivity profiling, corroborated by clinical experience, have identified much smaller spectra of resistant mutations for second-generation TKIs (Table 2) and these spectra are essentially non-overlapping (with the exception of the T315I mutation, as anticipated above). Hence, BCR-ABL1 kinase domain mutation screening is recommended in patients who fail TKI therapy, since detection of specific mutations influences the choice of the second- or subsequent-line TKI [79]. Ponatinib was rationally designed to bind mutant BCR-ABL1 as effectively as it binds native BCR-ABL1. Indeed, it is the only currently available option for T315I-positive patients [80]. Anecdotal reports, however, suggest that under the selective pressure of ponatinib, the T315I may further change into T315M or T315L [81, 82].

**Table 2:** List of the most frequent BCR-ABL1 kinase domain mutations resistant to ATP-competitive inhibitors reported in published studies

imatinib						nilotinib	dasatinib	bosutinib	ponatinib
M237V	L273M	F311L	E355D/G	V379I	A397P	Y253F/H	V299L	V299L	T315M
M244V	E275K/Q	**T315I**	F359V/I/C	A380T	S417F/Y	E255K/V	**T315I**	**T315I**	T315L
L248R	D276G	F317L/V/I/C	D363Y	F382L	I418S/V	**T315I**	F317L/V/I/C	?	
G250E/R	T277A	F359V/I/C	L364I	L384M	S438C	F359V/I/C			
Q252R/H	E279K	Y342H	A365V	L387M/F	E453G/K				
Y253F/H	V280A/I	M343T	L370P	M388L	E459K/V				
E255K/V	V289A	A344V	V371A	Y393C	P480L				
E258D	V299L	M351T	E373K	H396R/P	F486S				

The T315I is highlighted in bold and underlined. The question mark indicates that finding novel resistant mutations in the near future cannot be excluded

Sequencing of TKIs in patients who fail multiple lines of therapy has more recently brought up the issue of compound mutations. A compound mutant arises when two mutations are acquired by the same BCR-ABL1 molecule, thus by the same clone, as opposed to polyclonality where two clones acquire a single mutation each (Additional file 3: Figure S3). The term ‘compound mutant’ was coined at the very dawn of the second-generation TKI era – when dasatinib treatment of some imatinib-resistant patients was found to result in the acquisition of dasatinib-resistant mutations by BCR-ABL1 molecules already harbouring imatinib-resistant mutations [83]. Double compound mutants are by far the most frequent; compound mutants with three or even four mutations may also, occasionally, be detected – but too many mutations seem to be poorly tolerated [84, 85]. Detection of compound mutants might have important clinical implications. According to two recent studies, the IC_50_ values of second-generation TKIs and of ponatinib experimentally derived for many compound mutants are much higher than those each single mutant would exhibit [86, 87]. Such in vitro data suggest that i) the great majority of compound mutants are likely to be highly resistant to all second-generation TKIs; ii) some compound mutants might be challenging even for ponatinib. Very recently, a study in mice has predicted mutations interfering with asciminib binding. Such mutations (A337V, P465S, V468F, I502L) hit different residues as compared to those detected in case of resistance to ATP-competitive inhibitors, hence the hypothesis that combining both inhibitory modes might prevent mutation-driven resistance [70].

**Fig. 5:**
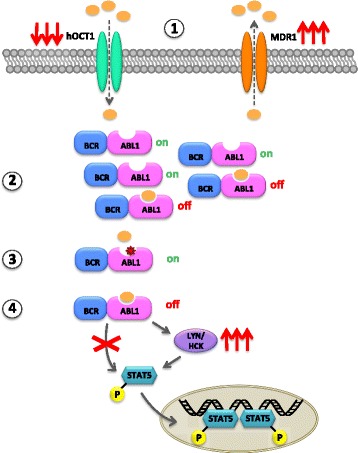
Overview of the mechanisms of resistance to BCR-ABL1 inhibition. According to the currently available data obtained in patients and/or cell lines, resistance may be due to (1) overexpression/increased activity of the efflux pump MDR1, and/or downmodulation/decreased activity of the influx pump hOCT1. This may result also from gene polymorphisms; (2) gene amplification and/or BCR-ABL1 mRNA and protein overexpression to levels that cannot be inhibited by achievable plasma concentrations of the TKI; (3) point mutations in the BCR-ABL1 kinase domain that interfere with TKI binding; (4) activation of alternative/downstream signaling pathways, e.g. of the SRC family kinases. Resistance mechanisms are not necessarily mutually exclusive

Kinase domain mutations are the most extensively studied mechanism of TKI resistance (mainly because of its actionability), but they are neither the only nor even the most frequent one (Fig. 5) [88]. Little, however, is known about other mechanisms, that have been investigated only in cell line models or in very small subsets of patients. In the pivotal study by Sawyer’s group, 3 patients who were negative for the T315I mutations were found to carry multiple copies of the *BCR-ABL1* gene by fluorescence in situ hybridization analysis and a 4–20-fold increase in *BCR-ABL1* transcript levels [73]. This mechanism, most frequent in advanced phase patients, can be overcome by the more potent second-generation TKIs. BCR-ABL1-independent mechanisms have also been reported or hypothesized to occur in imatinib-resistant patients. Activation of compensatory pro-survival/anti-apoptotic pathways may play a role. In this regard, overexpression or hyperactivation of some members of the SRC family of kinases (LYN, HCK), key effectors downstream of BCR-ABL1, have been described in cell lines and in some imatinib- and nilotinib-resistant patients [89–92]. This was one of the *rationales* that prompted the clinical development of dasatinib and bosutinib, dual SRC/ABL1 inhibitors. More recently, other molecules have been implicated in BCR-ABL1-independent TKI resistance and evaluated as therapeutic targets in in vitro studies: FOXO1 [93], β-catenin [94], STAT3 [95], the nucleocytoplasmatic transport molecules RAN and XPO1 [96], Cobll1 and NF-κB signalling [97], the AXL tyrosine kinase [98]. However, it is premature to tell whether these recent findings will translate into more effective therapeutic strategies for resistant patients.

Primary resistance (i.e., upfront failure to achieve a satisfactory response to therapy, as opposed to relapse after an initial response) has been linked to altered expression levels and/or function of the transporter molecules responsible for imatinib influx/efflux. Efflux proteins like the P-glycoprotein (Pgp or MDR1) encoded by the *ABCB1* gene, have been shown to play a role in some in vitro studies [99, 100]. Certain *ABCB1* polymorphisms have also been reported to predict for response to imatinib [101–103], although there is not complete concordance among different studies, most probably because of the heterogeneity in patient populations and of the relatively small sample sizes. The expression and function of the human organic cation transporter 1 (hOCT1), mediating imatinib uptake, have also been linked to differences in response rates in imatinib-treated patients [104, 105]. For some second-generation TKIs like dasatinib and nilotinib, transport in and out of cells is known not to rely on these molecules, which explains why the limited efficacy of imatinib may be overcome by switching to another drug [106, 107].

It is also well established that CML stem cells are intrinsically insensitive to TKIs, mainly because they do not require BCR-ABL1 kinase activity for their survival. CML stem cells thus survive TKI therapy and represent a dangerous reservoir from which resistance/relapse may originate. In addition, stem cell persistence is thought to be (one of) the reason(s) why treatment-free remission may not be pursued in approximately half of the cases. Several molecules and pathways have been identified in an attempt to eradicate CML stem cells (extensively reviewed in [108]), but very few combinations of TKI plus drugs targeting such molecules/pathways have so far progressed from preclinical to clinical testing.

Last but not least, it is important to remember that in many cases, a sudden increase in disease burden as assessed by *BCR-ABL1* transcript level measurement, or even a relapse, must be ascribed not to a biological rissue but to patient nonadherence to therapy [109–112]. Compliance represents a major problem for all chronic, self-administered treatments. Although CML is a life threatening disease if not properly treated, and although TKIs are generally well tolerated, patients’ perception regarding the importance of regular TKI assumption and regarding the burden of adverse reactions may be very different from physicians’ perception. This results in non-intentional or even in intentional lack of compliance, which may have serious consequences if not timely identified and addressed.

## Conclusions

The BCR-ABL1 fusion protein is probably the most extensively studied oncogenic tyrosine kinase and it is certainly the first that could successfully be targeted therapeutically. Being it the only genetic hit in CML pathogenesis or not, turning off BCR-ABL1 kinase activity with TKIs results in stable and ‘profound’ responses in terms of logarithmic reduction of detectable *BCR-ABL1* transcripts – so that some patients can nowadays discontinue the treatment and can be considered ‘functionally cured’. Nevertheless, the majority of newly diagnosed CML patients will have to face the perspective of life-long TKI treatment. As in all cancers, tumor escape mechanisms have been observed – mainly the acquisition of point mutations impairing TKI binding, fostered by the high genetic instability of leukemic cells – but proper choice and sequencing of the five TKIs currently available for first- or second-/subsequent-line treatment of CML patients enables to prevent or to counteract resistance in the majority of cases. Although the search for novel inhibitors and inhibitory approaches continues (also in an attempt to eradicate CML stem cells), the focus is now shifting to nonbiological issues, like how to maximize patient compliance to chronic treatment and how to manage the economic burden of such treatment, only partially mitigated by the recent patent loss by imatinib.

## Additional files

Additional file 1: Figure S1. Rare *BCR-ABL1* transcripts. (PDF 135 kb)

Additional file 2: Figure S2. Cartoon representation of the ABL1 kinase domain in complex with the five ATP-competitive inhibitors currently approved for the treatment of CML and with the allosteric inhibitor asciminib. (A-­E) Imatinib, nilotinib and ponatinib are type 2 inhibitors and bind to the inactive conformation of the kinase. Dasatinib and bosutinib are type 1 inhibitors and bind to the active conformation of the kinase. (F) ABL1 in complex with asciminib (ABL001 or GNF-­5; indicated with an arrow) and nilotinib. See text for details on binding modes. Images obtained with the web-­based 3D viewer NGL (PDB entries: 2HYY [imatinib]; 3CS9 [nilotinib]; 2GQG [dasatinib]; 3VE4 [bosutinib]; 3OXZ [ponatinib]: 5MO4 [asciminib and nilotinib]). (PDF 1965 kb)

Additional file 3: Figure S3. Difference between compound and polyclonal mutations. The red and green stars indicate two distinct mutations, that may be acquired by the same BCR-ABL1 molecules (compound) or by distinct BCR-ABL1 molecules. (PDF 663 kb)

## Abbreviations

ABL1: Abelson 1 gene
BCR: Breakpoint Cluster Region
CML: chronic myeloid leukemia
Ph: Philadelphia
TKI: tyrosine kinase inhibitor
Ph +: Philadelphia chromosome-positive
Ph-: Philadelphia chromosome-negative
B-ALL: B-cell acute lymphoblastic leukemia
SH: SRC Homology
CP: chronic phase
AP: accelerated phase
BP: blastic phase
α-IFN: alpha interferon
NGS: next generation sequencing
ATP: adenosine triphosphate
A-loop: activation loop
P-loop: phosphate-binding loop
PDB: Protein DataBase
