# Supplementary figures and images for: Chronic myeloid leukemia: the paradigm of targeting oncogenic tyrosine kinase signaling and counteracting resistance for successful cancer therapy
Source: Mol Cancer. 2018 Feb 19;17:49. doi: 10.1186/s12943-018-0780-6 (PMC5817796; doi:10.1186/s12943-018-0780-6)

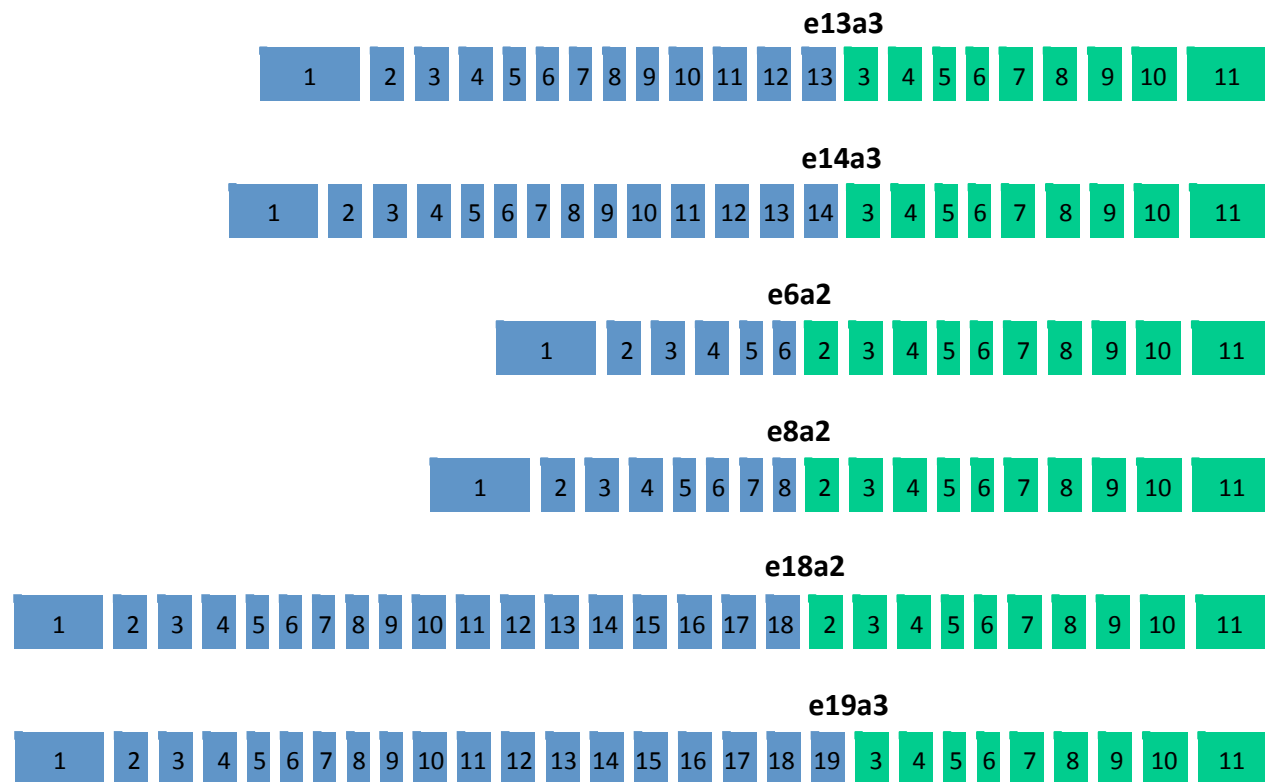

**Figure S1** – Rare *BCR-ABL1* transcripts

Supplement: Supplementary file 1 — Rare BCR-ABL1 transcripts. (PDF 135 kb) [file 12943_2018_780_MOESM1_ESM.pdf]
