# Supplementary material for: Chronic myeloid leukemia: the paradigm of targeting oncogenic tyrosine kinase signaling and counteracting resistance for successful cancer therapy
Source: Mol Cancer. 2018 Feb 19;17:49. doi: 10.1186/s12943-018-0780-6 (PMC5817796; doi:10.1186/s12943-018-0780-6)
Supplement: Supplementary file 2 — Cartoon representation of the ABL1 kinase domain in complex with the five ATP-competitive inhibitors currently approved for the treatment of CML and with the allosteric inhibitor asciminib. (A-­E) Imatinib, nilotinib and ponatinib are type 2 inhibitors and bind to the inactive conformation of the kinase. Dasatinib and bosutinib are type 1 inhibitors and bind to the active conformation of the kinase. (F) ABL1 in complex with asciminib (ABL001 or GNF-­5; indicated with an arrow) and nilotinib. See text for details on binding modes. Images obtained with the web-­based 3D viewer NGL (PDB entries: 2HYY [imatinib]; 3CS9 [nilotinib]; 2GQG [dasatinib]; 3VE4 [bosutinib]; 3OXZ [ponatinib]: 5MO4 [asciminib and nilotinib]). (PDF 1965 kb) [file 12943_2018_780_MOESM2_ESM.pdf]

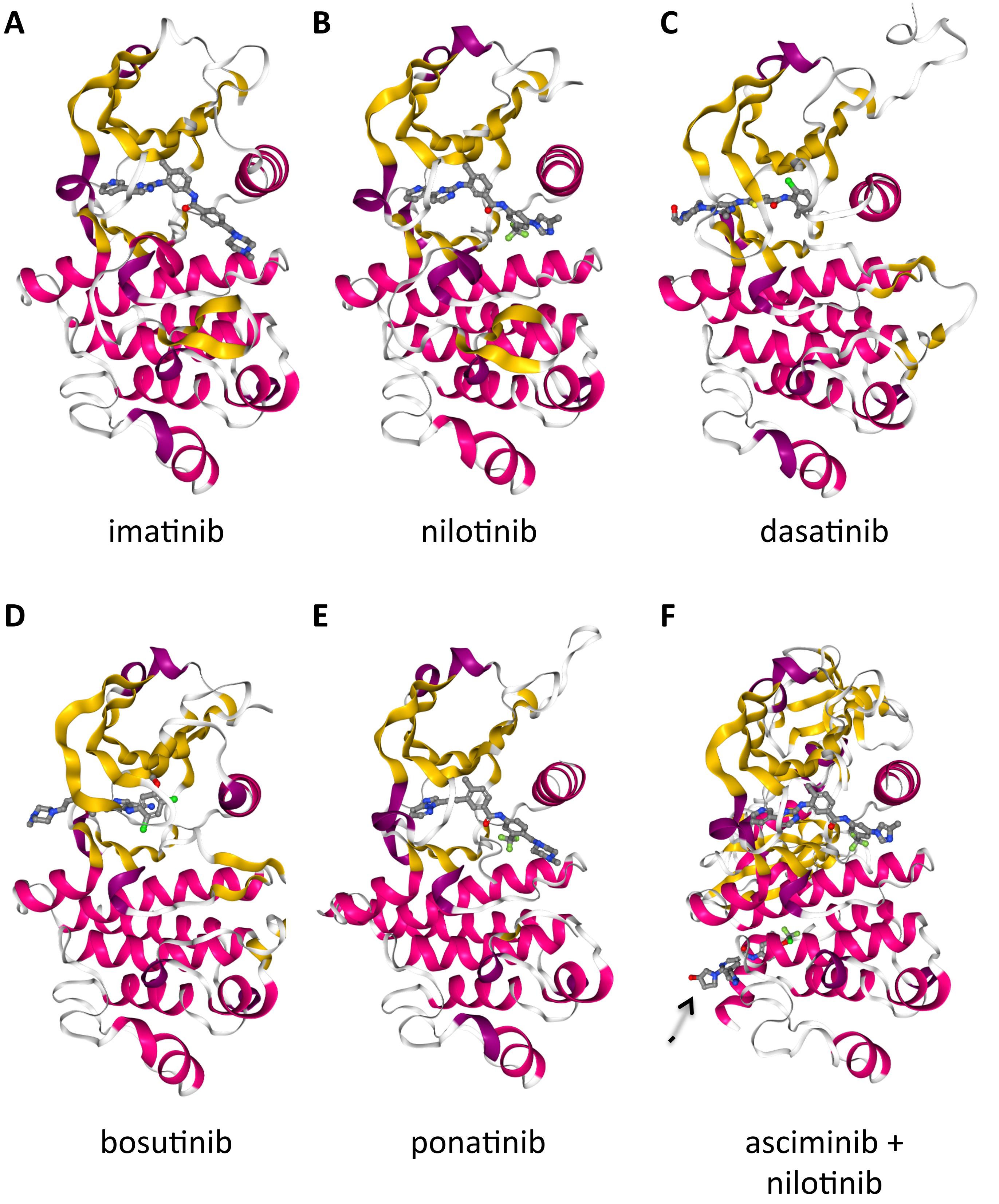

Figure S2 – Cartoon representation of the ABL1 kinase domain in complex with the five ATP-competitive inhibitors currently approved for the treatment of CML and with the allosteric inhibitor asciminib. (A-E) Imatinib, nilotinib and ponatinib are type 2 inhibitors and bind to the inactive conformation of the kinase. Dasatinib and bosutinib are type 1 inhibitors and bind to the active conformation of the kinase. (F) ABL1 in complex with asciminib (ABL001 or GNF-5; indicated with an arrow) and nilotinib. See text for details on binding modes. Images obtained with the web-based 3D viewer NGL (PDB entries: 2HYY [imatinib]; 3CS9 [nilotinib]; 2GQG [dasatinib]; 3VE4 [bosutinib]; 3OXZ [ponatinib]: 5MO4 [asciminib and nilotinib]).
