# Supplementary material for: Chronic myeloid leukemia: the paradigm of targeting oncogenic tyrosine kinase signaling and counteracting resistance for successful cancer therapy
Source: Mol Cancer. 2018 Feb 19;17:49. doi: 10.1186/s12943-018-0780-6 (PMC5817796; doi:10.1186/s12943-018-0780-6)
Supplement: Supplementary file 3 — Difference between compound and polyclonal mutations. The red and green stars indicate two distinct mutations, that may be acquired by the same BCR-ABL1 molecules (compound) or by distinct BCR-ABL1 molecules. (PDF 663 kb) [file 12943_2018_780_MOESM3_ESM.pdf]

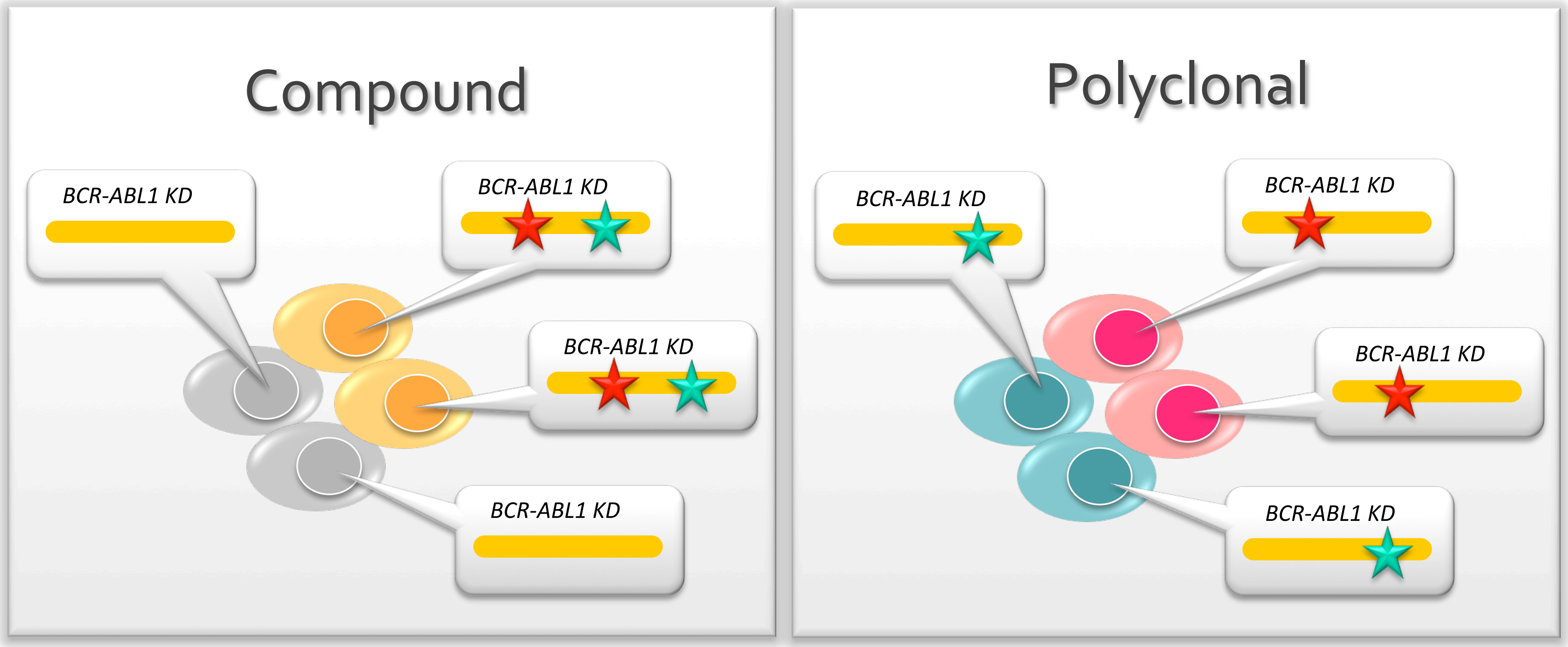

Supplementary Figure 3 – Difference between compound and polyclonal mutations. The red and green stars indicate two distinct mutations, that may be acquired by the same BCR-ABL1 molecules (compound) or by distinct BCR-ABL1 molecules
